# Phenotype-dependent effects of EpCAM expression on growth and invasion of human breast cancer cell lines

**DOI:** 10.1186/1471-2407-12-501

**Published:** 2012-10-30

**Authors:** Agnieszka Martowicz, Gilbert Spizzo, Guenther Gastl, Gerold Untergasser

**Affiliations:** 1 Laboratory of Experimental Oncology, Tyrolean Cancer Research Institute, Innsbruck, Austria; 2 Day Hospital of Haematology and Oncology, Franz Tappeiner Hospital, Merano, Italy; 3 Laboratory of Tumor Biology and Angiogenesis, Department of Internal Medicine V, Innsbruck Medical University, Innsbruck, Austria

**Keywords:** EpCAM, Lentivirus, Xenografts, RNA interference, Epithelial to mesenchymal transition

## Abstract

**Background:**

The epithelial cell adhesion molecule (EpCAM) has been shown to be overexpressed in breast cancer and stem cells and has emerged as an attractive target for immunotherapy of breast cancer patients. This study analyzes the effects of EpCAM on breast cancer cell lines with epithelial or mesenchymal phenotype.

**Methods:**

For this purpose, shRNA-mediated knockdown of EpCAM gene expression was performed in EpCAM^high^ breast cancer cell lines with epithelial phenotype (MCF-7, T47D and SkBR3). Moreover, EpCAM^low^ breast carcinoma cell lines with mesenchymal phenotype (MDA-MB-231, Hs578t) and inducible overexpression of EpCAM were used to study effects on proliferation, migration and *in vivo* growth.

**Results:**

In comparison to non-specific silencing controls (n/s-crtl) knockdown of EpCAM (E#2) in EpCAM^high^ cell lines resulted in reduced cell proliferation under serum-reduced culture conditions. Moreover, DNA synthesis under 3D culture conditions in collagen was significantly reduced. Xenografts of MCF-7 and T47D cells with knockdown of EpCAM formed smaller tumors that were less invasive. EpCAM^low^ cell lines with tetracycline-inducible overexpression of EpCAM showed no increased cell proliferation or migration under serum-reduced growth conditions. MDA-MB-231 xenografts with EpCAM overexpression showed reduced invasion into host tissue and more infiltrates of chicken granulocytes.

**Conclusions:**

The role of EpCAM in breast cancer strongly depends on the epithelial or mesenchymal phenotype of tumor cells. Cancer cells with epithelial phenotype need EpCAM as a growth- and invasion-promoting factor, whereas tumor cells with a mesenchymal phenotype are independent of EpCAM in invasion processes and tumor progression. These findings might have clinical implications for EpCAM-based targeting strategies in patients with invasive breast cancer.

## Background

The epithelial cell adhesion molecule (EpCAM, CD326) is a transmembrane glycoprotein originally discovered as a colon carcinoma-associated antigen [1]. The glycosylated transmembrane protein consists of a 289 amino acid extracellular domain (EpEX) and a short 26 amino acid intracellular domain (EpICD) [2]. EpCAM localizes to the basolateral membrane in normal polarized epithelia, but in carcinoma this expression pattern changes to an intense uniform membranous overexpression that is frequently associated with cytoplasmic staining [3]. In addition, EpCAM is hyperglycosylated to a 40 kDa or 42 kDa isoform in carcinoma tissue as compared with healthy autologous epithelia [4,5]. In breast carcinoma, strong EpCAM expression is observed in less differentiated tumors [6] and associates with larger tumors, nodal metastasis and poorer overall survival [6-8]. Moreover, in breast carcinoma EpCAM has been reported to be upregulated in large metastases as compared with the matched primary tumor [9]. Strong EpCAM expression has been shown to be a poor prognostic factor in both node-positive [8,9] and node-negative disease [6].

For invasion into stromal or adipose tissue breast cancer cells need to develop a mesenchymal phenotype [10]. This change in cell morphology is known as epithelial to mesenchymal transition (EMT) and seems to require downregulation of tight junction proteins, EpCAM and E-cadherin followed by re-expression at the site of the future metastasis [11]. In breast cancer, EMT has been estimated to occur in nearly 18% of tumors *in vivo*[12]. Under these conditions, EMT is defined as the occurrence of a variable proportion of tumor cells that express mesenchymal markers, such as vimentin and tenascin [13,14].

EpCAM downregulation has already been associated with EMT [15,16]. Small metastases in mice with colon carcinoma were EpCAM-negative. In contrast, large metastases in the same mouse displayed a level of expression equal to that of the primary tumor, a finding that possibly reflects the re-expression at the metastatic site [17].

This study analyzed different types of breast carcinoma cells derived from metastasis for their expression of EpCAM. Based on EpCAM protein expression levels we used lentiviral systems to knock down or induce EpCAM gene expression. Knockdowns were done in epithelium-like EpCAM^high^ cell lines (T47D, MCF-7, and SkBr3) and inducible overexpression was performed in EpCAM^low^ cell lines (Hs578t, MDA-MB-231) that display a mesenchyme-like phenotype. Our data demonstrate that EpCAM^high^ cancer cells with epithelial morphology clearly benefit from EpCAM overexpression with regard to proliferation and capacity to invade host tissue. In contrast, *in vitro* proliferation of EpCAM^low^ mesenchymal breast cancer cell lines is independent of EpCAM. Furthermore, we provide evidence that *in vitro* migration of tumor cells with mesenchymal phenotype is weakly inhibited by overexpression of EpCAM. Similar observations were made *in vivo*, where EpCAM-overexpressing mesenchymal tumor cells showed reduced invasive growth *and* more cell infiltrates of the innate immune system.

## Methods

### Cell lines

MCF-7, T47D and SkBR3 cell lines were purchased from the American Type Culture Collection (ATCC). All three cell lines were isolated from metastasis and display an epithelium-like phenotype. MCF-7, T47D cell lines were cultivated in MEM, Eagle’s medium (PAA Laboratories GmbH), and SKBR 3 in McCoy’s medium (PAA Laboratories GmbH) containing 10% fetal calf serum (PAA Laboratories GmbH) and 100 IU/mL penicillin, 100 μg/mL streptomycin and 2 mM glutamine (all PAA Laboratories GmbH). MCF-7^ns-ctrl^, MCF-7^E#2^, T47D^ns-ctrl^, T47D^E#2^, SkBR3^ns-ctrl^ and SkBR3^E#2^ cell lines were generated by lentiviral transfection with the corresponding pGIPZ shRNA mir virus and selected with 3Âµg/mL puromycin (Invitrogen) for 5 days in standard culture medium.

Hs578t and MDA-MB-231 cells (ATCC) originate from metastasis of human mammary carcinosarcoma and a poorly differentiated adenocarcinoma, respectively. Moreover, the latter cell type is an elongated and a highly invasive, metastatic phenotype. Hs578t and MDA-MB-231 cells were cultivated in RPMI1640 containing 10% tetracycline-free FCS (Clontech). Parental lines of Hs578t and MDA-MB-231 were lentivirally transfected (pLENTI6/TR, Invitrogen) and selected with 1 mg/mL neomycin (Biochrom) to express the tetracycline repressor (TR) protein. Selected Hs578t^TetR^ and MDA-MB-231^TetR^ lines were lentivirally transfected (pLenti6.3 EpCAM) and stable cell lines selected with 2 μg/mL blasticidin (Invitrogen). Hs578t^TetR^^EpCAM^ and MDA-MB-231^TetR EpCAM^ lines were propagated in 10% tetracycline-free FCS (Clontech).

Immortalized non-tumorigenic human mammary epithelial cells (MCF-10A) were obtained from the ATCC and cultivated Dulbecco's modified Eagle's medium F12 (DMEM/F12) supplemented with 5% horse serum (both Invitrogen), 1% penicillin/streptomycin (all PAA Laboratories GmbH), 0.5 μg/mL hydrocortisone, 10 μg/mL insulin and 20 ng/mL recombinant human EGF (all Sigma Biochemicals). Human Mammary Epithelial Cells (HMECs) were purchased from Promocell. HMECs were cultivated in Mammary Epithelial Cell Growth Medium with recommended supplements (Promocell, 0.004 mL/mL Bovine Pituitary Extract, 10 ng/mL epidermal growth factor, 5 μg/mL insulin and 0.5 μg/mL hydrocortisone) on collagen type I (Sigma Biochemicals) -coated ventilated plastic flasks.

### Immunohistochemistry

Tissue sections were deparaffinized and hydrated in xylene and graded alcohol series. Antigen retrieval was performed in a water bath (95°C) for 20 min with a target retrieval solution (Dako Cytomation), and endogenous peroxidase activity was blocked with 3% H_2_O_2_/methanol. Sections were incubated in blocking solution containing 10% fetal calf serum (Dako Cytomation) for 45 min and then stained for one hour with primary antibody (mouse anti-human EpCAM, ESA, clone VU-1D9, Novocastra, 1 μg/mL). Moreover, serial sections were incubated with an alpha smooth muscle cell actin (clone 1A4; 1:100, Sigma Biochemicals) or Ki67 (clone MIB-1, Dako Cytomation), or desmin (clone CD33, Dako), or vimentin (clone V9, Dako), or E-cadherin (clone 32A8, Cell Signaling Technology). Primary antiserum was detected after incubation with a biotinylated secondary antibody (biotinylated rabbit anti-mouse IgG, Vector Laboratories Inc.) using the Vectastain Elite ABC Kit (Vector Laboratories Inc.) and the FAST DAB Tablet Set (Sigma Biochemicals). Sections were counterstained with Meyer’s hematoxylin and mounted with Pertex (Medite).

### Immunofluorescence

Cells were seeded on eight-well culture chamber slides (Falcon BD Labware) at densities of 20,000 cells/well and incubated overnight in 5% CO_2_ at 37°C. After being fixed in 4% paraformaldehyde and permeabilized with 0.2% Triton-X-100 cells were blocked with PBS containing 3% BSA for 45 min at room temperature (RT). E-cadherin- (clone 32A8, Cell Signaling Technology), vimentin- (clone v9, Dako Cytomation), and cytokeratin 18- (clone DC 10, Dako) specific antibodies were applied in a 1:200 dilution at RT for two hours. After washing in PBS cells were incubated with secondary fluorochrome-labeled antibodies (FITC-labeled rabbit anti-mouse, Dako Cytomation) and nuclei were counterstained with DAPI (Molecular Probes). Cells were embedded in fluorescent mounting medium (Dako Cytomation) and viewed with constant laser settings using a fluorescence microscope (Zeiss Axiovert 200M with Axiovision 4.7 Software, Carl Zeiss Optics).

### Western Blot analysis

Cells were harvested and lysed in an RIPA buffer (Sigma) containing protease inhibitors (Complete Mini EDTA-free; Roche Applied Science). Total protein (20 μg) was denaturated, separated by a 4%-20% SDS-PAGE (Criterion TGX, Bio-Rad) and transferred to Immuno-Blot^TM^ polyvinylidene difluoride (PVDF) membrane (Bio-Rad). After blocking the membrane in 5% non-fat milk powder dissolved in phosphate-buffered saline (PBS), membranes were incubated overnight in 0.5% non-fat milk powder at 4°C with primary mouse antibodies. Antibodies used for Western Blot analysis were a mouse monoclonal directed against EpCAM (C-10; Santa Cruz Biotechnology) and a mouse monoclonal against alpha tubulin (clone B5-1-2; Sigma Biochemicals). Afterwards, membranes were incubated for one hour with an HRP-conjugated goat anti-mouse IgG (Dako Cytomation) diluted 1:1,000. After washing, a chemoluminescent substrate (LumiGLO Reagent and Peroxide, Cell Signaling Technology) was added to the membrane, which was then exposed in the Chemidoc XRS station (Biorad Laboratories). Different glycosylated isoforms of EpCAM were analyzed by Western Blot after deglycosylating the protein in RIPA buffer by means of PNGase F and Endo H (both from New England Biolabs). Band intensities were analyzed and calculated using the Quantity One 4.6 software (BioRad Laboratories).

### Generation of lentiviral particles for knockdown and overexpression of EpCAM

Three lentiviral transfer vectors (pGIPZ shRNAmir) targeting different regions of the EpCAM open reading frame (clone Ids: V2LHS_134162, V2LHS_134158, V2LHS_23265) and validated non-silencing control (ns-ctrl; RHS4346) were ordered from Open Biosystems. pGIPZ clones were propagated in the presence of 25 μg/ml zeocin (Invitrogen) and plasmids purified with the high-speed Plasmid MIDI Kit (Qiagen). Lentiviral particles were produced by transfecting HEK 293FT cells (Invitrogen) with helper plasmid mix (trans-lentiviral packaging system, Open Biosystems) and Lipofectamin 2000 (Invitrogen).

The EpCAM cDNA (NM_002354, Open Biosystems) was subcloned into the Gateway pENTR11dual section vector (Invitrogen). The pENTR11 vector was site-specifically recombined with the pDEST6.3 vector (Invitrogen) using the Gateway LR Clonase II Pus Enzyme Mix (Invitrogen). The resulting pDEST6.3 vector with the EpCAM open-reading frame was transformed and propagated in One-Shot Stabl3 bacteria (Invitrogen). Lentiviruses were produced in HEK 293FT cells (Invitrogen) by transfecting cells with the pDEST6.3 EpCAM vector and helper plasmid mix (ViraPower, lentiviral support kit, Invitrogen) using Lipofectamine 2000. Lentiviral titers were determined by real time PCR and quantification of *woodchuck hepatitis virus posttranscriptional response element* expression (WPRE-for: 5-actgacaattccgtggtgtt; WPRE-rev: 5-agatccgactcgtctgagg), as described elsewhere [18].

### Quantitative RT-PCR analysis

Total RNA was isolated from all cell lines using the TriReagent (Sigma Aldrich), according to the manufacturer’s instructions. Thereafter, viral and genomic DNA in the RNA samples was digested with the RQ1 DNAse (Promega). The cDNA was amplified from 1 μg total RNA using the SuperScript II Reverse Transcriptase Kit (Invitrogen Life Technologies). For validation, real time RT-PCR was performed using a SensiMix SYBR No-ROX Kit (Bioline) and a Rotor-Gene 6000 detection system (Corbett Research). Primers were designed to amplify human-specific GAPDH (for: 5-ctgacctgccgtctagaaaa; rev: 5-gagcttgacaaagtggtcgt), TATA-Box binding protein (for: 5- ggagccaagagtgaagaaca; rev: 5-agcacaaggccttctaacct) and *EpCAM* (for: 5-gctggtgtgtgaacactgct; rev: 5-acgcgttgtgatctccttct).

### Real time cell proliferation and migration assay (xCelligence system)

Real time cell proliferation and migration experiments were performed using the RTCA DP instrument (Roche Diagnostics GmbH), which was placed in a humidified incubator maintained at 5% CO_2_ and 37°C. For proliferation assays cells were seeded in complete medium in 16-well plates (E-plate 16, Roche Diagnostics GmbH) at a density of 5,000 cells/well. The plate, which contained gold microelectrodes on its bottom, was monitored every ten minutes for four hours (adhesion process), then once every 30 min until the end of the experiment, (72h). Cell migration was performed using special 16-well plates with 8-μm pores (CIM plate 16, Roche Diagnostics GmbH). These plates, resembling conventional transwells, have microelectrodes on the underside of the membrane, which is located between an upper and a lower chamber. Cells were seeded into the upper chamber at a density of 20,000 cells/well in a serum-free medium. Lower chambers were filled with complete medium. The plate was monitored every 15 min for 12 h. Data analysis was performed using the RTCA software 1.2 supplied with the instrument (Roche Diagnostics).

### 3D tumor collagen plug formation

The ice-cold collagen type I solution was obtained by mixing on ice 8.2 volumes of rat-tail collagen type I (2.0 mg/ml, Sigma Aldrich), 1 volume of MEM 10x concentrated medium and 0.8 volumes of sterile 0.2 M NaOH; pH was adjusted to 7.4 with sterile NaOH. 3D collagen drop cultures were generated by resuspending 5.0 × 10^5^ breast cancer cells in 30 μl collagen mix. Hs578t^TetR EpCAM^ and MDA-MB-231^TetR EpCAM^ cells were additionally stimulated with tetracycline (1 μg/mL). Then 30 μl collagen drops were dropped into a sterile 10-cm, Ø cell culture dish (Falcon) coated with sterilized parafilm and allowed to polymerize for 30 min at 37°C in a humidified cell culture incubator. After polymerization of 3D cultures, collagen plugs were either transferred into 96-well plates (1 plug/well) and maintained in a 200 μL standard complete cell culture medium for 3D *in vitro* cell proliferation experiments or directly grafted on a chicken CAM for *in vivo* tumor growth studies.

### 3D proliferation assay

MCF-7^ns/ctrl^, MCF-7^E#2^, T-47D^ns/ctrl^, T-47D^E#2^, SkBR3^ns/ctrl^, SkBR3^E#2^ or Hs578t^TetR EpCAM^ or MDA-MB-231^TetR EpCAM^ were grown in 96-well plates in the presence of 2.5% serum. Hs578t^TetR EpCAM^ or MDA-MB-231^TetR EpCAM^ was cultivated in complete medium with or without 1 μg/mL tetracycline. After three days of *in vitro* growth a cell proliferation assay was performed (BrdU ELISA kit, GE Healthcare). Cells were pulsed with 5-bromo-2’-deoxyuridine 20 h prior to measurement in ELISA. Microplates with cell plugs were centrifuged at 2,000 g to allow the plugs to attach to the bottom of the plate. Thereafter, cells in plugs were fixed with the fixative solution and the ELISA performed according to the manufacturer instructions.

### Chicken chorioallantoic membrane (CAM) tumor xenograft model

On embryo development day 0 fertilized chicken eggs (Gallus domesticus) were placed in a 75%-80% humidified 37°C incubator to allow normal embryo development. On day 3 eggs were opened, egg shells removed and embryos were placed in a sterile Petri dish in an egg incubator (Grumbach) to induce CAM development. Embryos were inspected daily until the day of experiment. On day 8, when the chorioallantoic membrane (CAM) and its vasculature were well developed, the experiment was performed. Subsequently, four onplants (collagen cell plugs) per chicken were grafted onto the CAM. Growth of tumor xenografts was inspected on a daily basis using a stereo fluorescence microscope (Olympus SZW 10). On day 6 post-grafting chicken embryos were sacrificed by hypothermia, xenografts/CAM cut out and stored in 4% paraformaldehyde (Sigma Biochemicals) for immunohistochemistry or in TRI reagent (Sigma Biochemicals) for RNA isolation.

### Statistical analyses

Statistical analyses were performed with the GraphPad Prism™ software 5.0 (GraphPad Software, Inc.). All tests of statistical significance were two-sided. Student’s *T* test and the Mann–Whitney *U* Test were used to study differences between two groups. Statistical analyses of quantitative PCR data were performed according to the delta Ct method described by Pfaffl *et al.*[19] and p values were calculated with Student's *T* test or the two-way ANOVA test.

## Results

### Expression of EpCAM in breast cancer cell lines and primary human mammary epithelial cells

**Figure 1:**
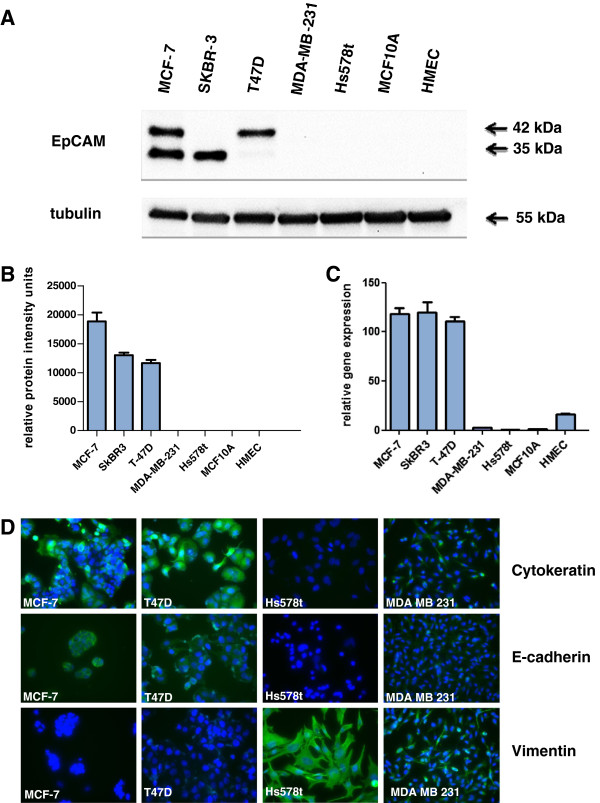
**Expression of EpCAM in human breast cancer cell lines and primary epithelial cells. (A)** Protein expression was analyzed by Western Blot analysis with an antibody directed against the extracellular domain of EpCAM. Primary cells (HMECs) showed weak or no expression of EpCAM. Epithelial tumor cells displayed strong EpCAM expression, whereas EpCAM expression decreased in more mesenchymal tumor cells. The two different bands represent glycosylated and basic isoforms of EpCAM. Tubulin served as internal loading control. **(B)** Densitometric analysis of EpCAM protein expression of two independent experiments. Values indicate relative intensity units. **(C)** EpCAM gene expression was analyzed by real-time PCR analysis using TATA-Box binding protein and GAPDH as internal housekeeping genes. All cell lines were analyzed in triplicate and normalized to the expression levels of MCF-10A cells. **(D)** Tumor cell lines were phenotyped by immunofluorescence analysis after staining for epithelial markers cytokeratin-18 and E-cadherin and the mesenchymal marker vimentin. Magnification 400x.

EpCAM gene and protein expression was analyzed in various breast cancer cell lines, immortalized (MCF-10A) and normal mammary epithelial cells (HMECs). Normal and immortalized cells showed no detectable protein in Western Blot analysis (Figure 1A/B). The same holds true for the mesenchyme-like breast cancer cell lines MDA-MB-231 and Hs578t (Figure 1A/B). By contrast, epithelium-like tumor cell lines MCF-7, T47D, and SkBR3 showed strong expression of the EpCAM protein as basic and glycosylated isoforms of 35 and 40 KDa (Figure 1A). Western Blot results were confirmed by real-time PCR (Figure 1C). Epithelial breast carcinoma cell lines had significantly stronger EpCAM gene expression than did HMECs or immortalized MCF-10A mammary epithelial cells. Mesenchymal MDA-MA-231 and Hs578t displayed low amounts of EpCAM mRNA (Figure 1C). Based on these findings EpCAM^high^ and EpCAM^low^ breast cancer cell lines were phenotyped for markers of epithelial or mesenchymal differentiation. MCF-7 and T47D cells with strong EpCAM expression were strongly positive for epithelial markers cytokeratin 18 and E-cadherin and negative for mesenchymal vimentin expression as determined by immunofluorescence analysis (Figure 1D). In contrast, Hs578t and MDA-MB-231 cells with low EpCAM expression were strongly positive for the mesenchymal marker vimentin and negative or weakly positive for E-cadherin and cytokeratin 18. Hs578t cells were more transdifferentiated to mesenchymal cancer cells and more fibroblast-like than MDA-MB-231 (Figure 1D).

### Knockdown of EpCAM inhibited cell proliferation of epithelium-like EpCAM^high^ breast cancer cells *in vitro*

**Figure 2:**
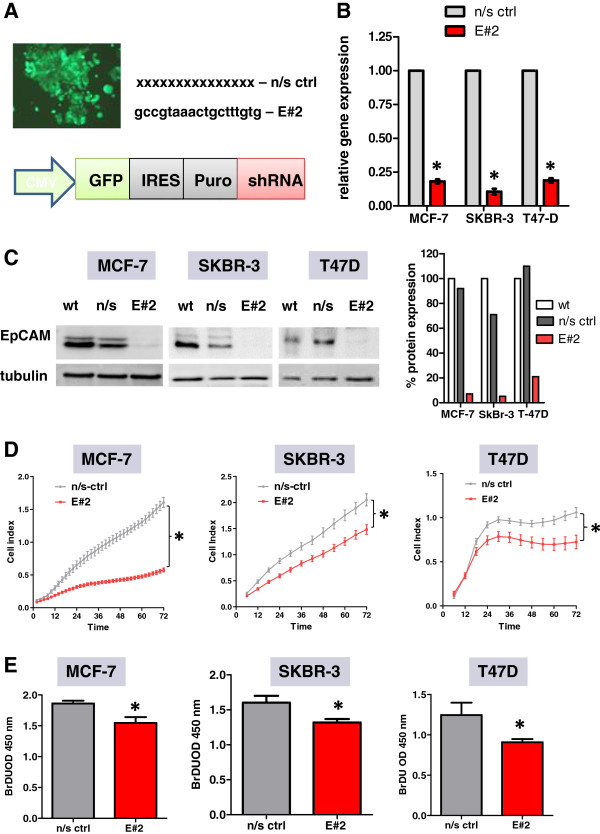
**Lentiviral knockdown of EpCAM gene expression by shRNA in EpCAM**^**high **^**human breast cancer cell lines. (A)** Lentiviruses were generated that express not only the green fluorescent protein (GFP) and the puromycin resistance gene (Puro), but also shRNAs targeting EpCAM (E#2) or a non-silencing control sequence (n/s ctrl). Transfected cells expressed turbo GFP and were selected with puromycin for five days. **(B)** The efficacy of EpCAM knockdown (E#2) in comparison to that of non-silencing controls (n/s ctrl) was proven by real-time PCR. Gene expression was reduced below 20% of the control value in all three cell lines analyzed. All cell types were analyzed in triplicate. **(C)** Knockdown of EpCAM was proven on the protein level by Western Blot analysis. Tubulin alpha served as internal loading control. In comparison to wild-type cells (wt) and n/s ctrl, EpCAM expression decreased in cells expressing E#2. Densitometric analysis of two independent experiments (means) as compared to wild type expression (100%). **(D)** Proliferation of transfected cells was monitored in real-time by the xCelligence system over a period of 72 h. E#2-expressing cells displayed significantly less proliferation and a smaller cell number when cultivated under serum-reduced conditions (n=6). **(E)** 3D growth of transfected cells was studied in collagen plugs by incorporating BrdU and subsequently measuring BrdU in a specific ELISA. E#2-expressing cells showed a significantly lower rate of DNA synthesis (n=6). Stars indicate p values < 0.05.

Based on our previous analysis of EpCAM expression in epithelium- and mesenchyme-like breast carcinoma cell lines we selected three EpCAM^high^ cell lines for lentiviral knockdown studies, i.e. T47D with a strongly glycosylated, MCF-7 with basic and glycosylated, SkBR-3 with only basic EpCAM isoform (Figure 1A). Glycosylation patterns were analyzed after deglycosylating all EpCAM isoforms by means of endoglycosidases to a 35 kDa not glycosylated isoform (data not shown). EpCAM-high cell lines were lentivirally transfected to express a non-silencing control (n/s ctrl) or EpCAM-specific shRNA together with turbo GFP and a puromycin resistance gene (Figure 2A). Three different sequences, E#1, E#2 and E#3 targeting the open reading frame (ORF) of EpCAM, were pretested (data not shown) and the E#2 sequence giving the best knockdown was used for further studies. Effective knockdown was controlled by real-time PCR on all three cell lines (Figure 2B) and by Western Blot analysis (Figure 2C). Lentivirally transfected cells were selected for five days with puromycin and subsequently used for analysis of cell proliferation. In comparison to n/s ctrl cells, E#2 knockdown cells displayed a significantly lower capacity to proliferate *in vitro* after seeding the same starting amount of cells into the xCelligence real-time cell counting system (Figure 2D). These observations were made in all three cell lines under serum-reduced conditions, i.e. 2.5% serum, over the observed time period of three days. The pro-survival effect of EpCAM was less pronounced under full mitotic conditions, i.e. 10% serum in the culture medium (data not shown). Additionally, cell proliferation was analyzed by measuring DNA synthesis, i.e. BrdU incorporation, under 3D culture conditions in collagen drops with tumor cells. All used cell lines displayed a significant reduction in DNA synthesis after knockdown of EpCAM within three days of *in vitro* growth (Figure 2E).

### Knockdown of EpCAM inhibits invasion of epithelium-like EpCAM^high^ breast cancer cell xenografts

**Figure 3:**
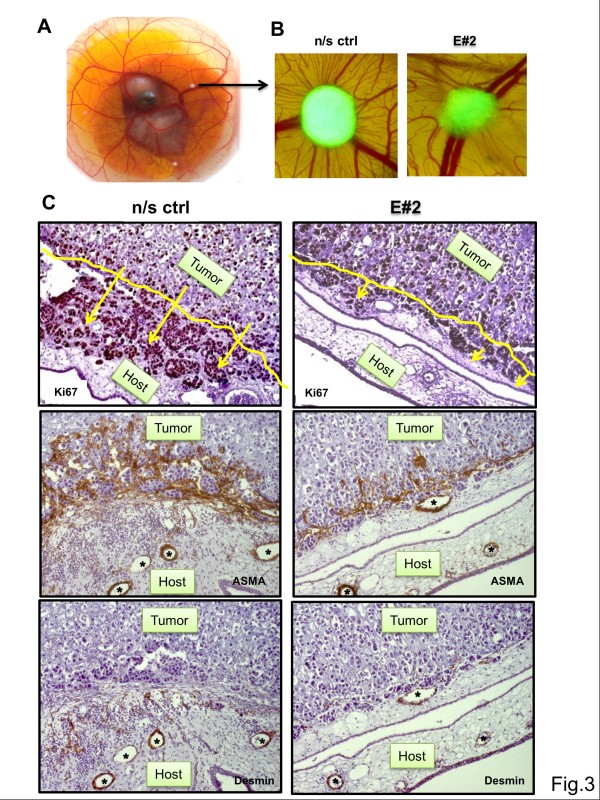
**Effects of lentiviral knockdown of EpCAM on *in vivo *growth and invasion of EpCAM**^**high **^**breast cancer cell lines. (A)***In vivo* growth of MCF-7 breast cancer cell xenografts in the chicken chorioallantoic membrane model (CAM). Magnification 1x. **(B)** Xenografts form tumors (arrow) that can be monitored due to expression of GFP by stereo-fluorescence microscopy. Within six days of observation tumors grew inside the CAM and attracted and co-opted blood vessels. Magnification 20x. **(C)** Immunohistochemical analysis of tumor xenografts in the host tissue. Brown color indicates a positive reaction. All nuclei were counterstained with hematoxylin (blue). Proliferating tumor cells were stained for the nuclear antigen Ki67, mural cells of blood vessels (myofibroblasts) with alpha smooth muscle cell actin (ASMA) or pericytes (fibroblasts) with desmin. Knockdown of EpCAM (E#2) resulted in tumors with less invasion and proliferation in host tissue and in a reduction of stromal responses to the tumor. Yellow arrows indicate invading tumor cell clusters and yellow lines borders between xenograft and host tissue. (Magnification 200x). *In vivo* experiments were performed with at least 12 embryos per group and 4 onplants/CAM. Asterisks indicate blood vessels of the underlying CAM.

Based on our *in vitro* results we aimed to study the effects of EpCAM knockdown in the *in vivo *situation. For this purpose, we selected the chicken chorioallantoic membrane (CAM) xenograft model that allows us to study human cells in an animal microenvironment [20,21]. Chicken embryos have only innate immune responses and tolerate growth of human cancer cells for a period of six days when cells are transplanted on the surface of the CAM tissue (Figure 3A). MCF-7 and T47D onplants can be observed daily by stereo-fluorescence microscopy due to expression of turbo GFP (Figure 3B). SkBr3 cells failed to form vascularized tumors that invade the CAM (data not shown). Macroscopically, MCF7 and T47D tumors did not differ in size when analyzing and calculating only surface areas on CAMs. Immunohistochemical analysis of tumor cells by the proliferation marker Ki67 revealed that knockdown of EpCAM significantly affected tumor cell invasion into host tissue (Figure 3C). Knockdown of EpCAM strongly reduced the invasion capacity of MCF-7 and T47D tumor cells in chicken tissue (Figure 3C, yellow line and arrows). Moreover, as determined by staining for mesenchymal cells, such as pericytes (desmin) and smooth muscle cells of chicken blood vessels (ASMA), reactive responses of the chicken stroma to the invading tumor cell front were nearly abolished (Figure 3C).

### Overexpression of EpCAM does not support proliferation or migration of mesenchyme-like EpCAM^low^ breast cancer cells *in vitro*

**Figure 4:**
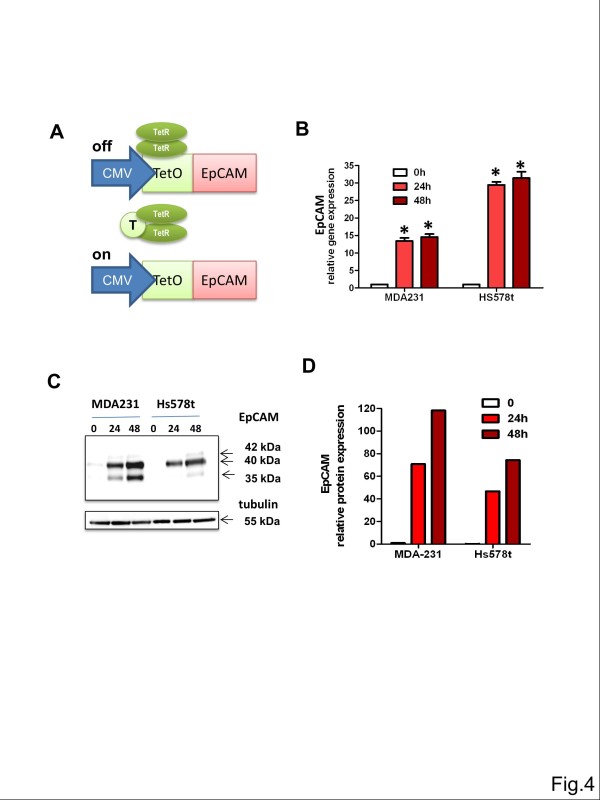
**Inducible overexpression of EpCAM in EpCAM**^**low **^**human breast cancer cell lines with mesenchymal phenotype. (A)** EpCAM^low^ cell lines MDA-MB-231 and Hs578t were transfected to express the tetracycline-repressor protein (TetR). A lentiviral construct for tetracycline (T)-inducible expression of EpCAM was generated and tumor cells stably integrating the construct were selected and named Hs578t^TetR^^EpCAM^ or MDA-MB-231^TetR EpCAM^. **(B)** Stimulation with tetracycline for 24 h resulted in a strong induction of EpCAM gene expression as determined by real-time PCR. Mean±SEM of three independent experiments. **(C)** Moreover, upregulation of EpCAM was observed on the protein level as determined by Western Blot analysis. In addition to the unglycosylated protein, EpCAM was produced as a glycosylated and hyperglycosylated isoform in both cell lines analyzed. **(D)** Densitometric analysis of EpCAM protein expression of two independent experiments. All means are calculated in comparison to expression of MDA-MB 231 cells before induction with tetracycline.

**Figure 5:**
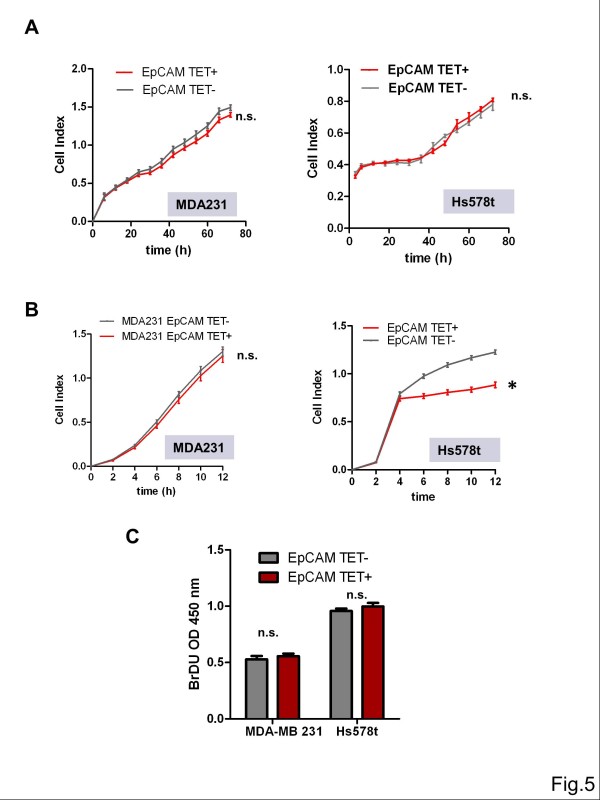
***In vitro *growth and migration of EpCAM**^**low **^**human breast cancer cell lines after overexpression of EpCAM. (A)** Cell numbers were determined by the xCelligence real-time system after addition of tetracycline and incubation for three days (n=4). EpCAM overexpression (EpCAM TET+) did not support *in vitro* cell proliferation under serum-reduced culture conditions. **(B)***In vitro* cell migration was assessed with the transwell CIM plate in the xCelligence real-time migration system 24 h after addition of tetracycline (n=4). MDA-MB-231^TetR EpCAM^ cells showed no significant (n.s.) changes in cell migration after overexpression of EpCAM (EpCAM TET+). In contrast, more mesenchymal Hs578t^TetR EpCAM^ were significantly inhibited by overexpression of EpCAM (EpCAM TET+). **(C)** Cell proliferation was studied in 3D collagen plug cultures after addition of tetracycline and incubation for three days by measuring the incorporation of BrdU into synthesized DNA. Overexpression of EpCAM (EpCAM TET+) had no significant (n.s.) impact on DNA synthesis *in vitro* (n=4). * indicates p value < 0.05.

Based on our previous analysis on EpCAM expression in epithelium- and mesenchyme-like breast carcinoma cell lines (Figure 1) we selected two EpCAM^low^ cell lines for inducible lentiviral overexpression studies, i.e. MDA-MB-231 and Hs578t breast carcinoma cells, both with a mesenchymal cancer phenotype (Figure 1D). Cells were lentivirally transfected to express the bacterial tetracycline-repressor protein (TET-R) and selected with neomycin for ten days. Thereafter, MDA-MB-231^TetR^ and Hs578t^TetR^ cell lines were lentivirally transfected with an EpCAM open reading frame under a tetracycline-repressible CMV promoter (Figure 4A) and short-time selected with blasticidin. Both cell lines, MDA-MB-231^TetR EpCAM^ and Hs578t^TetR EpCAM^, were tested for tetracycline-induced expression of EpCAM by real-time PCR (Figure 4B). In comparison to cells growing without tetracycline and having low endogenous transcript levels, addition of tetracycline induced a 20- to 30-fold expression of the EpCAM gene. In addition to gene expression, we also analyzed the induction of EpCAM protein expression by Western Blot analysis (Figure 4C). In both cell lines, MDA–MB-231^TetR EpCAM^ and Hs578t^TetR EpCAM^, we observed a strong induction of EpCAM protein already 24 h after adding tetracycline to the culture medium (Figure 4D). It is noteworthy that both cell lines produced predominantly the glycosylated (40 kDa) isoform and, to a minor extent, also the hyperglycosylated isoform (42 kDa). Tetracycline-inducible EpCAM cell lines were used for analysis of *in vitro* cell proliferation in the xCelligence system. Induction of EpCAM by tetracycline had no statistically significant effect on cell proliferation within an observed time period of three days (Figure 5A). Since Hs578t as well as MDA-MB-231 cells show a high capacity to migrate, we studied the effects of EpCAM on *in vitro* cell migration. Cells were pre-incubated for 24 h with tetracycline, counted and then transferred into the xCelligence real-time cell migration system. Induction of EpCAM by tetracycline did not affect *in vitro* cell migration within an observed period of 12 h in MDA-MB-231 cells (Figure 5B). In contrast, the fibroblast-resembling Hs578t cells were significantly inhibited in cell migration after overexpression of EpCAM (Figure 5B). Analyses of DNA synthesis in the BrdU incorporation assay of 3D collagen drop cultures also did not reveal significant differences in *in vitro* cell proliferation, three days after induction of EpCAM (Figure 5C).

### EpCAM downregulates E-cadherin gene expression in mesenchyme-like EpCAM^low^ breast cancer cells

**Figure 6:**
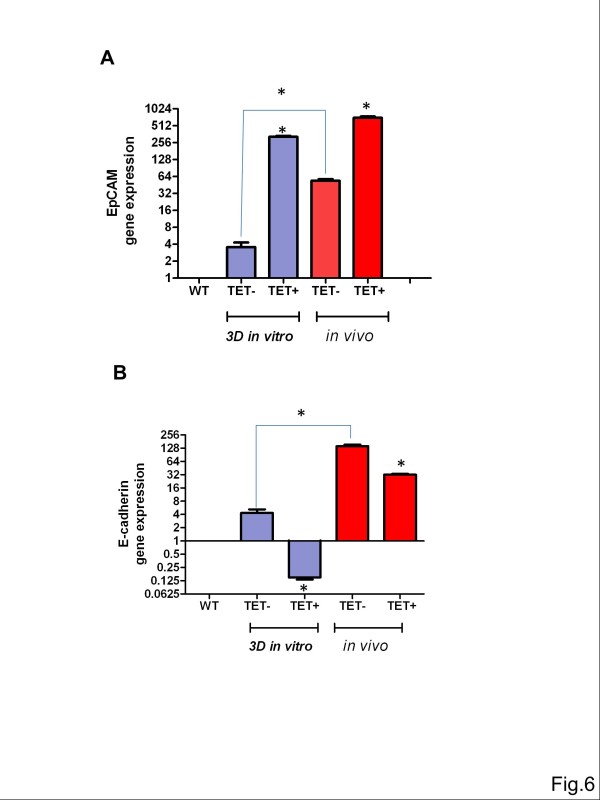
***In vivo *growth induces EpCAM and E-cadherin gene expression.** MDA-MB-231^TetR EpCAM^ cells were grown *in vitro* in 3D culture in collagen or *in vivo* in the chicken CAM. EpCAM **(A)** and E-cadherin **(B)** gene expression was quantified relative to the low expression levels in the parental cell line MDA-MB-231 (WT). RNA was isolated 24 h after stimulation with tetracycline (TET+, n=4) or without tetracycline (TET-, n=4) and analyzed by means of RT-qPCR. Xenografts (n=4/group) were grown for five days in the chicken embryo before isolation and analysis of RNA. Note, *in vivo* growth induced EpCAM and E-cadherin expression in MDA-MB-231 cells. EpCAM overexpression inhibited induction of E-cadherin gene expression in MDA-MB-231 cells. * indicates p value ≤0.05.

Depending on cell culture conditions and the respective microenvironment, MDA-MB 231 cells with parentally low EpCAM and E-cadherin expression can induce endogenous transcription of both genes (Figure 6A/B). The MDA-MB-231 cell line, when growing under 3D culture conditions, displayed larger amounts of EpCAM and E-cadherin than under standard 2-D culture conditions. Transcript levels of E-cadherin and EpCAM were even more elevated under *in vivo* culture conditions in the CAM.

Interestingly, tetracycline-induced overexpression of EpCAM in MDA-MB-231 cells was responsible for downregulation of E-cadherin gene expression, namely under 3D conditions and in the CAM *in vivo*.

### MDA-MB-231 xenografts with EpCAM overexpression display reduced invasion into host tissue and strong innate immune responses

**Figure 7:**
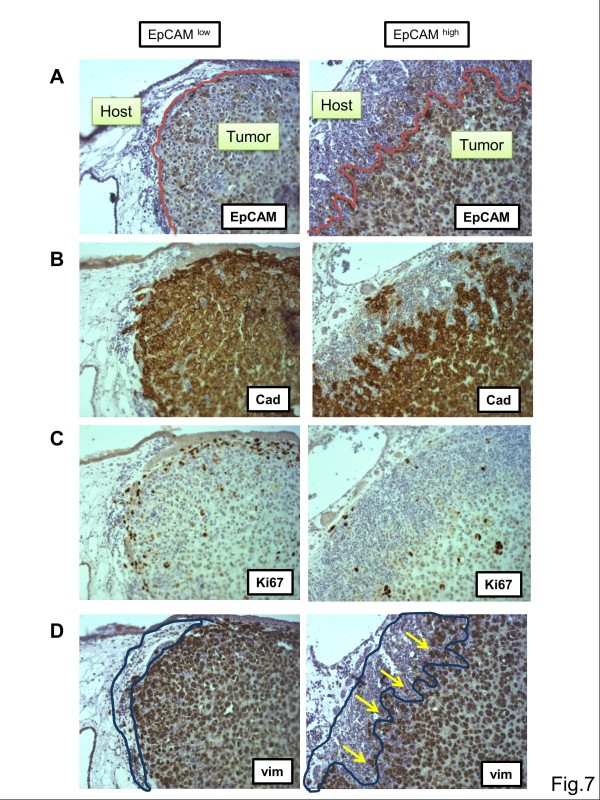
**Overexpression of EpCAM in MDA-MB-231 xenografts.** MDA-MB-231^TetR EpCAM^ cells were grafted into chicken embryos and *in vivo* growth analyzed after five days in the presence and absence of tetracycline. *In vivo* experiments were performed with at least 12 embryos per group and 4 onplants/CAM. **(A)** Cross-sections of tumor xenografts in host tissue were analyzed by immunohistochemistry for EpCAM protein. Tumor invasion front into host tissue is indicated by a red line. **(B)** Tumor cells were stained for E-cadherin expression to indicate specifically human tumor cells. **(C)** Proliferating human tumor cells were stained for the expression of Ki67. **(D)** Mesenchyme-like human tumor cells were stained for the marker vimentin (vim). Note the massive clusters of chicken heterophils in EpCAM-high tumors (blue area) that incorporate between tumor cells in the invasion front (yellow arrows). Magnification: 100 x.

Hs578t^TetR EpCAM^ cells could not be used in the chicken CAM xenograft model, because they did not invade CAM tissue, lacked neovascularization and displayed many apoptotic cells due to lack of oxygen and nutrients (data not shown). Consequently, experiments were performed with the highly invasive MDA-MB-231^TetR EpCAM^ cells. MDA-MB-231^TetR EpCAM^ cells have low endogenous EpCAM expression on the mRNA level, even without induction with tetracycline (Figure 6A). Stimulation with tetracycline in the collagen-graft resulted in an approx. 50-fold induction of EpCAM gene expression, as determined by real time PCR (Figure 6A). Interestingly, *in vivo* MDA-MB-231 cells re-express E-cadherin, and EpCAM overexpression represses E-cadherin gene expression (Figure 6B). Macroscopically, tumors did not differ in size after six days of *in vivo* growth (data not shown). We therefore performed immunohistochemical analysis of CAM sections to analyze changes on the cellular and tissue level (Figure 7A-D). MDA-MB-231^TetR EpCAM^ without EpCAM overexpression were highly invasive, growing deeply into host tissue and showed only dispersed cell clusters that were weakly positive for EpCAM (Figure 7A). By comparison, EpCAM-overexpressing tumors were clearly positive for EpCAM protein expression and displayed an irregular invasion front into host tissue (Figure 7A, red line). This front was also visualized by staining for human tumor cells using cadherin (Figure 7B). EpCAM-overexpressing tumors had no compact and homogeneous invasion front into host tissue. Moreover, when analyzing the proliferation marker Ki67 EpCAM^low^ tumors had a line of highly mitotic cells in the invasion front that was nearly lost in EpCAM^high^ tumors (Figure 7C). Inside tumors, the number of Ki67-positive cells did not differ significantly. Additionally, we stained mesenchymal human tumor cells for vimentin protein expression (Figure 7D). EpCAM^high^ tumors showed no differences in vimentin expression, suggesting that EpCAM overexpression did not directly affect epithelial to mesenchymal transition (EMT). Moreover, EpCAM^high^ tumors showed more innate immune responses from the chicken immune system. Heterophils (granulocytes) of the chicken were recruited to the tumor invasion front (blue line) and invaded in massive clusters between tumor cells (Figure 7D, yellow arrows).

## Discussion

EpCAM functions as a target in antibody-based clinical trials, and the European Medicines Agency (EMA) approved in 2009 the use of the trifunctional bispecific antibody catumaxomab. In human breast cancer patients, the EpCAM-specific antibody adecatumumab shows EpCAM-dependent activity in clinical studies [22,23]. These antibodies bind to EpCAM and enhance the immunological response to EpCAM-positive cancer cells, either in blood or malignant ascites [24]. Moreover, EpCAM has emerged as a promising target structure on cancer stem cells [25]. Cancer stem cells expressing EpCAM are more tumorigenic than EpCAM-negative stem cells [11,26] and, due to their resistance to radiation and chemotherapy, targeting EpCAM might be a promising approach to impede tumor recurrence after chemo- or radiotherapy.

**Figure 8:**
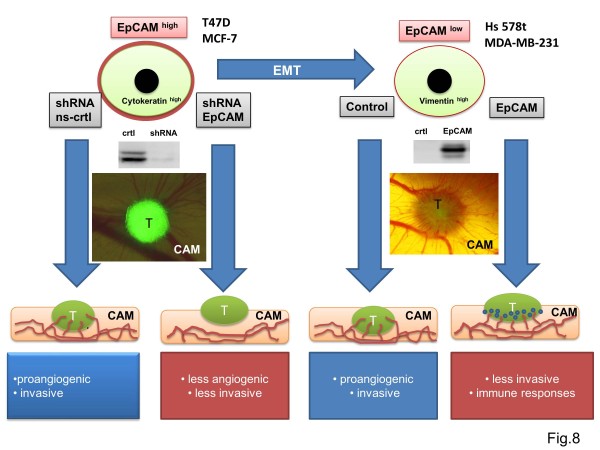
**Hypothetical model of the role of EpCAM in breast cancer.** EpCAM function might switches from bad to good depending on the phenotype of cancer cells, i.e. their status of epithelial to mesenchymal transition. More epithelium–like cancer cells with strong cytokeratin and E-cadherin expression (T47D, MCF-7) need EpCAM to support proliferation, invasion and tumor angiogenesis. EpCAM knockdown results in fewer invasive and angiogenic tumors in the chicken chorioallantoic membrane (CAM) xenograft model. GFP-positive tumor cells (T) can be monitored on the CAM in real-time and obtain support via the blood vessels (red lines). In contrast, more mesenchyme-like tumor cells, having high vimentin expression, grow independently of EpCAM. Overexpression of EpCAM in these cells disturbs invasion and tumor angiogenesis and prompts strong innate immune responses by the chicken host (blue cells indicate heterophils).

Whether EpCAM has a pro- or an anti-tumorigenic function in cancer appears to be dependent on the cancer type [11]. An ‘ugly’ role of EpCAM is reflected by studies describing both a protective and a promoting role within the very same cancer type [11]. Tumor conglomerates are very heterogeneous and contain distinct cell types. Therefore, the role of EpCAM seems to depend less on the carcinoma type, and more on the respective tumor microenvironment [11]. Thus, EpCAM can shift from a protective to a promoting role in cancer, depending on the differentiation or dedifferentiation status of epithelial cancer cells. The complex and hypothetical model of action of the EpCAM molecule is summarized in Figure 8.

This study analyzed different breast cancer cell lines, all deriving from breast cancer lymph node metastasis. In particular, it analyzed the effects of EpCAM expression on cell lines according to their phenotype, i.e. the status of epithelial to mesenchymal transition (EMT). According to their EpCAM expression profile we used lentiviral systems to alter endogenous gene expression and studied the effects on *in vitro* and *in vivo* tumor growth. Particularly, in breast carcinoma cells with high cytokeratin-18 and E-cadherin expression, i.e. epithelial phenotype, EpCAM displays growth- and invasion-promoting effects. Our data are in line with reports on pharyngeal, lung and breast cancer cells, where EpCAM seems to support tumor progression [2,11]. Indeed, in epithelial/acinar tumor cell lines with high cytokeratin expression, such as the FaDu hypopharynx carcinoma cells, EpCAM overexpression supports proliferation and cell cycle by modifying Wnt signaling [27]. Moreover, a recent study by Maghazal *et al.* showed that EpCAM signaling is important during embryogenesis [28]. Independently of its putative cell adhesion function, EpCAM strongly supports cell movements and mixing of tissues by a protein kinase C-dependent signaling mechanism.

Hs578t and MDA-MB-231 cells with a more mesenchymal phenotype do not gain survival benefits from EpCAM overexpression or signaling. These cells typically have low EpCAM and E-cadherin expression and display invasive growth and migration like reactive fibroblasts [10,29]. Our findings are in line with those of early studies by Litvinov and colleagues on fibroblast-like cells. They found that overexpression of EpCAM *in vivo* inhibited the invasive growth of fibroblastic L cells and dedifferentiated mammary carcinoma L153S cells. Moreover, EpCAM overexpression suppressed invasive colony growth of fibroblastic L cells in EHS matrigel *in vitro*[30]. Interestingly, EpCAM overexpression did not support mesenchymal-to-epithelial reverting transition (MErT). However, MDA-MB-231 cells started to re-express E-cadherin *in vivo* in our xenograft model, a fact already observed by Chao *et al.*[31]. This MErT process was not affected by overexpression of EpCAM. Xenografts still retained the high vimentin expression, but EpCAM-overexpressing tumors displayed massive infiltrations of chicken heterophils, which are avian analogs of mammalian neutrophils [32]. In addition, few monocytes/macrophages avian heterophils are recruited to areas of inflammation and to the invading tumor cell front, thereby strongly promoting angiogenesis [33]. Thus, EpCAM overexpression in mesenchyme-like tumors seems to stimulate inflammatory processes by activating and attracting neutrophils to the invasion front. Tumor-associated neutrophils (TANs) can promote the progression of primary tumors [34]. However, depending on TGF-β signaling TAN can also differentiate into a population with antitumor activity [35]. Recently, it was reported that neutrophils in tumor-bearing subjects can act to eliminate disseminated tumor cells and thus provide antimetastatic protection [36].

Most importantly, all these findings might even have a clinical impact since EpCAM-based targeting agents (i.e. catumaxomab) are now available for therapeutic use also in breast cancer patients [24]. In fact, breast cancer is a frequent cause of malignant ascites and especially invasive lobular breast cancer patients develop peritoneal carcinomatosis with malignant ascites [37]. However, EpCAM expression is usually low in lobular breast cancer as compared to ductal cancer [38], and the phenotype of lobular breast cancer resembles that of cancer cells undergoing EMT described in the present study. Considering our data on an anti-tumorigenic effect of EpCAM in mesenchyme-like cancer cells, targeting EpCAM in patients with lobular breast cancer might even result in a counterproductive effect. For this reason, particular attention should be given to clinical studies determining the efficacy of EpCAM-targeting agents in the subgroup of patients with lobular breast cancer.

## Conclusion

The role of EpCAM in breast cancer changes with tumor cell phenotype and the respective tumor microenvironment. Cancer cells with an epithelial phenotype use EpCAM overexpression and signaling for growth and invasion. In contrast, in tumor cells with a mesenchymal phenotype EpCAM expression decreases and the tumor cells grow independently of EpCAM signaling. Re-expression of EpCAM in these tumors inhibits migration and invasion and promotes inflammation and innate immune responses. Therefore, the fact that EpCAM has a dual role in tumorigenesis should be given consideration when planning EpCAM-targeted therapies in breast cancer patients.

## Competing interests

The authors declare that they have no competing interests.

## Authors’ contributions

AM established lentiviral constructs, transfected cell lines, carried out migration and proliferation assays, animal experiments and contributed substantially to the experimental design. GU planned experiments and drafted the manuscript. GS and GG participated in study design and data interpretation. All authors read and approved the final manuscript.

## Pre-publication history

The pre-publication history for this paper can be accessed here:

http://www.biomedcentral.com/1471-2407/12/501/prepub
